# The moderating role of conscientiousness in the temporal association of stress on sleep

**DOI:** 10.1111/jsr.14224

**Published:** 2024-04-29

**Authors:** Conny W. E. M. Quaedflieg, Camilla Bossi, Jessica Bruijel

**Affiliations:** ^1^ Department of Neuropsychology & Psychopharmacology, Faculty of Psychology and Neuroscience Maastricht University Maastricht The Netherlands; ^2^ Limburg Brain Injury Centre Maastricht The Netherlands

**Keywords:** conscientiousness, multi‐level modelling, neuroticism, sleep, stress, students

## Abstract

Personality traits have been associated with sleep problems and stress experience. However, their impact on objective sleep and the temporal relationship of stress on sleep has remained elusive. This study examined whether daytime stress predicts sleep the following night, and the moderating role of neuroticism and conscientiousness in this relationship. To introduce stress variability in natural daily stressors, we measured college students (*N* = 92) during exams (e.g. high academic stress) and at the start of new course period (e.g. low academic stress). Both objective (actigraphy) and subjective sleep, and daily self‐reported stress, were measured for 14 days and personality traits once. Reported daily stress was significantly higher in the exam period compared with baseline, suggesting that our natural manipulation did indeed result in variation in stress levels. Intra‐individual daily variations in stress were not associated with the following night's sleep timing, duration or fragmentation, implying that more stress during the day did not affect sleep the following night. Higher levels of neuroticism were associated with poorer daily subjective sleep quality and higher stress levels over the complete period. Neuroticism did not moderate the temporal association of stress on sleep. Conscientiousness moderated the association between intra‐individual stress and sleep fragmentation, and intra‐individual stress and wake‐up time. This implied that highly conscientious participants experienced less sleep fragmentation and woke‐up earlier after more stressful days. These results suggest an interconnected relationship among stress, sleep and personality. Focusing on one aspect, like handling stress or enhancing sleep quality, might yield positive effects on the rest.

## 1 INTRODUCTION

University students are a vulnerable group, who often experience high levels of stress (i.e. exam stress) during their studies (Gardani et al., 2022). Stress and stress regulatory systems have a major impact on sleep and the development of sleep disturbances. Multiple studies showed that higher stress levels were associated with a shorter sleep duration, more nocturnal awakenings, and decreased sleep efficiency (SE) and sleep quality using both subjective and objective measures (Astill et al., 2013; Doane & Thurston, 2014; Hanson & Chen, 2010). Even the anticipation of a future stressor, like an exam, can increase worry and cognitive arousal, and has a detrimental effect on sleep (Beck et al., 2022). These studies often averaged sleep variables over multiple days, and used trait stress measures ignoring the dynamic interplay between stress and sleep.

The few studies examining the temporal association between stress and sleep showed that stress directly affects the following night's sleep. Higher evening stress predicted a shorter sleep duration the upcoming night using objective sleep measures (e.g. actigraphy; Doane & Thurston, 2014; Yap et al., 2020). Increased stress levels raise the stress‐associated hormone cortisol, which impacts the sleep–wake cycle leading to earlier wake‐up time (Hirotsu et al., 2015). Furthermore, a shorter sleep time and lower SE measured with actigraphy were associated with higher next‐day stress, therefore creating a vicious cycle between stress and sleep. Inducing acute stress right before taking a 90‐min nap mainly impacted the first half of the nap possibly due to enhanced physiological arousal (Beck et al., 2022), while the anticipation of stressful events after sleep affected the second half of the nap possibly due to prolonged cognitive arousal. Many factors might moderate this relationship between stress and sleep, such as personality traits.

The traits most often associated with sleep are neuroticism and conscientiousness (Sutin et al., 2020). Neuroticism is defined as the tendency to experience negative emotions and distress, while conscientiousness is the tendency to be organized, disciplined and responsible (Digman, 1990). Individuals high in neuroticism and low on conscientiousness experience more stress‐related negative affect, worse physical and mental health, and are more prone to anxiety, which are factors known to disrupt sleep quality (Križan & Hisler, 2019; Leger et al., 2016; Strickhouser et al., 2017). Accordingly, both high neuroticism and low conscientiousness have been linked to sleep difficulties and poor sleep quality (Sutin et al., 2020). The few studies examining stress, sleep and personality in combination found that neuroticism and conscientiousness were associated with lower subjective sleep quality and experiencing more stress (Nédélec et al., 2021; Williams & Moroz, 2009). Furthermore, the association between personality and sleep was mediated by stress (Williams & Moroz, 2009). However, the effect of personality on objective sleep and the temporal dynamics between stress and sleep have remained elusive.

The present study therefore assessed whether daytime stress predicts sleep the following night, and the moderating role of neuroticism and conscientiousness in this relationship. Based on previous research showing a relationship between stress and sleep (Yap et al., 2020), we hypothesized that on days when participants experience higher stress levels, they would have a shorter total sleep time (TST), lower SE, higher wake after sleep onset (WASO), longer sleep‐onset latency (SOL) and worse subjective sleep quality. In addition, we hypothesized that the relationship between daily stress and worse sleep is stronger in participants with high neuroticism and low conscientiousness. To induce variability in stress levels, we assessed students during the exam period, thus using naturalistically occurring daily life stressors, and at the start of the new course period (e.g. low in academic stress). Both objective (i.e. actigraphy) and subjective sleep estimates, as well as subjectively experienced stress, were assessed daily for a 14‐day period. A better understanding of the relationship between stress and sleep and the role of neuroticism and conscientiousness could allow better identification and preventive interventions for individuals at risk of developing sleep disturbances under stress. This is particularly important considering that high levels of stress and sleep disturbances can have serious mental and physical health consequences both in the short‐ and long‐term (Kalmbach et al., 2018).

## 2 METHODS

### 2.1 Participants

Participants were college students (*N* = 92) that were recruited from Maastricht University through campus posting. The inclusion criteria were being a student, having an upcoming exam period (within 1–2 weeks) and being fluent in English. The exclusion criteria were a current diagnosis of a psychological disorder (e.g. depression or schizophrenia) or a sleep disorder (e.g. sleep apnea or insomnia), and use of medication that is known to affect sleep. Most of the participants were female (86%) and the mean age was 21 years (SD: 2.8; range of 17–37 years). Participants were recruited between October 2021 and November 2022.

### 2.2 Procedure

Participation started with a visit to the lab, participants provided informed consent, followed by collection of demographic data, and then the wrist actigraph was provided and explained. Participants were asked to wear the watch continuously on the non‐dominant wrist for a total of 14 days, starting 4–6 days before their final exam (exact date was provided) at 17:00 hours. Concurrent with wearing the watch, participants were asked to complete a diary each morning within 1 hr of waking up, where they provided information about the previous night and day (e.g. bedtimes, stress levels). After 14 days, participants answered questionnaires online about sleep, anxiety, personality and stress. Participants were rewarded with research points that students need to obtain as part of the requirements to fulfil the Bachelor of Psychology. The study protocol was approved by the Ethics Review Committee Psychology and Neuroscience of Maastricht University (ERCPN 242_124_09_2021). The study was registered at the open science framework (https://osf.io/rmf7c/?view_only=ea637e5e6162425c9e69cce4a1f1e989).

### 2.3 Measures

#### 2.3.1 Actigraphy

The GENEactiv movement sensor (Activinsights, Kimbolton, UK, MEMS sensor) was used to measure sleep. This non‐invasive watch‐like device monitors movement and thereby estimates sleep. The watch was set to 50 Hz, and the accompanying GENEactiv software was used to set‐up the watch and extract the data. The GENEactiv devices have good reliability and validity (Quante et al., 2018; te Lindert & Van Someren, 2013). Bedtime and wake‐up time from the sleep diaries were used to define the rest interval for the sleep analyses in matlab (te Lindert & Van Someren, 2013). Sleep outcome variables included bedtime, wake‐up time, TST, SE, SOL and WASO.

#### 2.3.2 Diaries

The Consensus Sleep Diary (CSD) was used to explore subjective sleep measure, and for the GENEactiv analyses to define the rest interval (Carney et al., 2012). The CSD includes questions about bedtime, lights off time, wake time, out of bedtime, self‐reported SOL, the number of and total time of WASO, TST, total time in bed, self‐reported sleep quality and satisfaction, and naps. Moreover, the diary asked about alcohol intake, caffeine intake, and drugs or medications that could have affected their sleep. For this study, questions about daily perceived stress were added to the CSD using visual analogue scales ranging from 1 (very relaxed) to 10 (very stressed; Lesage et al., 2012). Three scales were present, one regarding academic stress, one regarding non‐academic stress, and one about total stress experienced.

#### 2.3.3 Personality

Personality traits were assessed with the Big Five Inventory (BFI), which includes five dimensions: Openness; Conscientiousness; Extraversion; Agreeableness; and Neuroticism (John & Srivastava, 1999). The BFI consists of 44 items and has good psychometric properties (John & Srivastava, 1999).

#### 2.3.4 Sleep quality

Subjective sleep quality over the last month was assessed with the Pittsburgh Sleep Quality Index (PSQI; Buysse et al., 1989). This 19‐item questionnaire provides a global score between 0 and 21, with higher scores indicating poorer sleep quality. A score >5 indicates poor sleep quality. The PSQI has good psychometric properties (Buysse et al., 1989).

#### 2.3.5 Anxiety

Anxiety was assessed using the State–Trait Anxiety Inventory (STAI; Spielberger et al., 1983). This study only included trait anxiety, which consists of 20 items, with scores ranging between 20 and 80, and a score of >45 indicates high levels of anxiety. The STAI has good psychometric properties (Spielberger et al., 1983).

#### 2.3.6 Stress

The perception of stress was measured using the Perceived Stress Scale (PSS; Cohen et al., 1983). This 10‐item questionnaire provides a score between 0 and 40, with higher scores indicating more perceived stress. The PSS has good psychometric properties (Lee, 2012).

### 2.4 Data analysis

A priori power analyses indicated a sample of 85 participants (assuming a 75% completion rate) provided 85% power to detect a medium effect size of 0.25, using an alpha level of 0.05, for mixed model analyses (R package sjstats; Lüdecke & Lüdecke, 2019). In total, 100 participants were included in the study. One participant dropped out before wearing the wrist actigraph, two participants did not fill in the questionnaires and, of five participants, actigraphy data were missing due to malfunctioning of the actigraph, therefore 92 participants were included in the analyses. Days with outliers (>3SD from the mean) were removed from the data, leaving 840 days in the main data analyses.

Analyses were performed in R version 3.5.1 with the lme4 package (Bates et al., 2015). The assumptions of linearity and normal distribution of residuals of the models were tested. The residuals of SOL were not normally distributed, and this variable was therefore log transformed. For every model, effects sizes were calculated (e.g. conditional *R* ^2^, which is the portion of variance explained by fixed and random effects combined).

To explore whether the exam period induced stress, daily stress and sleep variables were compared between the exam period and baseline (start of the new courses/period) using paired sample *t*‐tests. For these analyses, sleep and stress variables were averaged for the period right before/during the exam(s) and baseline. Additionally, variability in sleep variables (standard deviation) between the exam period and baseline were compared using paired sample *t*‐tests. The days after the exam up to the Monday of the new period were not included in any of the analyses as we expect students to have poor sleep these days due to recovery from the exams and excessive alcohol use and partying. For comparison to previous research, the relationships between sleep and stress (averaged over the whole measurement period) and scores on the BFI, STAI, PSQI and PSS were assessed using Pearson correlations (these results are described in the supplementary materials).

To assess the intra‐individual associations of stress on sleep, and the moderation by personality traits, daily stress levels were centred at the person mean representing daily variations of stress (intra‐individual). Positive values indicated higher scores than the person's own average across time. Participants thus served as their own control, allowing thorough examination whether daily deviations from an individuals' average stress levels impacted sleep that night. Multilevel linear models with restricted maximum likelihood estimation method were used. Daily sleep variables (bedtime, wake‐up time, TST, SE, WASO, SOL and subjective sleep quality measured with CSD) were the dependent variables (*within‐subject*), and subsequent daily stress levels (*within‐subject*) and personality (neuroticism and conscientiousness; *between‐subject*) and their interaction were the independent variables, with daily alcohol and coffee consumption as covariates.

Similar exploratory analyses were performed to assess the bidirectional relationship between sleep and stress, that is, whether intra‐individual variation in the sleep predicted stress the next day. Daily stress levels were the dependent variable (*within‐subject*), and previous night sleep (bedtime, wake‐up time, TST, SE, WASO, SOL and subjective sleep quality; *within‐subject*) and personality (neuroticism and conscientiousness; *between‐subject*) and their interaction were the independent variables. A separate model was made for each sleep variable.

## 3 RESULTS

### 3.1 Participants

Demographics and scores on the questionnaires are presented in the supplementary materials Table S1. Poor sleep was common, with 46% of the participants reporting poor sleep quality (PSQI >5). Almost half of the participants reported symptoms of anxiety (48%, STAI >45). Missing data on the daily diaries was low, on average less than 1 day of the 14 days per subject was missing (mean 0.35, SD 1.05). Sleep variables averaged and their variability (SD) over the whole period per participant and their correlation with personality, stress and anxiety are shown in the supplementary materials (Table S2). Participants that reported more stress overall did also report worse subjective sleep quality (PSQI: *r* = −0.4, *p* ≤ 0.001) and in the daily diary (*r* = −0.3, *p* ≤ 0.001; Table S2).

### 3.2 Comparison between sleep and stress averaged over baseline and exam period

The daily sleep and stress variables averaged for the exam (4.6 days, SD: 1.0) and baseline (4.7 days, SD: 1.0) period are shown in Table 1. On average, participants reported higher daily total stress and worse sleep quality during the exam period compared with the baseline (all *p* < 0.001; Figure S1). Participants spent less time in bed during the exam period compared with baseline (*p* = 0.04). There were no differences in the timing of sleep, TST, SE, WASO or SOL between the exam period and baseline. There was more variability during the baseline period in bedtimes and wake‐up times compared with the exam period (Table S3). However, there was more variability in subjective sleep quality during the exam period compared with baseline. Moreover, participants used less alcohol and more caffeine during the exam period compared with baseline (all *p* < 0.01).

**TABLE 1:** Mean and standard deviations for sleep variables, stress and substance use for the baseline and exam period (*N* = 92).

Variables	Baseline		Exam period		Statistics
	Mean ± SD or *N* (%)	Range	Mean ± SD or *N* (%)	Range	
Actigraphy					
Number of days	4.6 ± 1.0	1.2–8	4.7 ± 1.0	1.0–6	*W* = 4543.5, *p* = 0.12
Bedtime (clock time 24 hr; hours)	00:51 ± 1.0	21:52–03:13	00:48 ± 1.1	21:36–03:14	*t*(1, 89) = −0.15, *p* = 0.88
Wake‐up time (clock time 24 hr; hours)	08:27 ± 1.0	05:03–11:12	08:19 ± 1.0	05:19–10:34	*t*(1, 89) = −1.28, *p* = 0.20
Time in bed (hr)	9.1 ± 1.1	5.0–11.8	8.9 ± 1.2	4.2–11.9	*t*(1, 89) = −2.06, *p* = 0.04*
TST (hr)	7.1 ± 0.9	3.2–9.7	7.0 ± 0.9	3.3–9.9	*t*(1, 89) = −1.56, *p* = 0.12
SOL (min)	14.4 ± 10.3	0.0–47.2	15.2 ± 12.0	0–67.5	*t*(1, 89) = 0.71, *p* = 0.48
SE (%)	88.2 ± 3.8	77.0–94.9	87.8 ± 4.1	74.2–97.3	*t*(1, 89) = −0.9, *p* = 0.40
WASO (min)	32.5 ± 11.5	11.3–65.5	33.1 ± 11.6	7.2–62.1	*t*(1, 89) = −0.16, *p* = 0.88
Self‐report					
Subjective sleep quality (daily sleep diary)	3.5 ± 2.0	0.5–5.0	3.3 ± 0.6	1.0–4.8	*t*(1, 89) = −4.20, *p* < 0.001***
Daily total stress	3.0 ± 1.6	0.0–8.2	6.5 ± 1.8	1.2–10.0	*t*(1, 89) = 17.54.20, *p* < 0.001***
Daily academic stress	1.8 ± 1.5	0.0–6.8	6.6 ± 2.1	0.2–10.0	*t*(1, 89) = 19.30.61, *p* < 0.001***
Alcohol use (%)	17.8 ± 20.1	0–100	8.1 ± 15.8	0–100	*t*(1, 89) = −4.44, *p* < 0.001***
Caffeine use (%)	47.5 ± 40.8	0–100	54.8 ± 42.7	0–100	*t*(1, 89) = 3.19, *p* = 0.002**
Medication/drugs use (%)	7.1 ± 18.3	0–100	8.1 ± 21.4	0–100	*t*(1, 89) = 0.60, *p* = 0.55

*Note*: Usage of alcohol, caffeine, medication and drugs is represented as the percentage of days on which the substance was consumed during that period.

Abbreviations: SE, sleep efficiency; SOL, sleep‐onset latency; TST, total sleep time; WASO, wake after sleep onset.

*p < 0.05; **p < 0.01; ***p < 0.001.

### 3.3 Intra‐individual associations of stress on sleep and the moderation by personality traits

After checking for outliers (>3SD from the mean), 840 observations clustered in 92 participants were included in the multilevel models. Table 2 presents the results of the multilevel models examining the temporal relationship of intra‐individual stress on sleep and the moderation by personality.

**TABLE 2:** Results from multilevel models of intra‐individual association between stress, sleep and the moderation by personality, while adjusting for daily alcohol and caffeine intake.

	Bedtime		Wake‐up time		TST		SE		WASO		SOL		Sleep quality	
	*R* ^2^ conditional		*R* ^2^ conditional		*R* ^2^ conditional		*R* ^2^ conditional		*R* ^2^ conditional		*R* ^2^ conditional		*R* ^2^ conditional	
Full model	0.49	*T* = 370.09, *p* < 0.001	0.35	*T* = 15.66, *p* < 0.001	0.33	*T* = 9.87, *p* < 0.001	0.34	*T* = 31.08, *p* < 0.001	0.40	*T* = 4.63, *p* < 0.001	0.15	*T* = 3.91, *p* < 0.001	0.22	*T* = 11.35, *p* < 0.001
Predictors	Beta	CI (95%)	Beta	CI (95%)	Beta	CI (95%)	Beta	CI (95%)	Beta	CI (95%)	Beta	CI (95%)	Beta	CI (95%)
Intra‐stress	−0.04	−0.09 – 0.01	−0.10	−0.15 – −0.04	0.06	−0.12 – −0.00	0.01	−0.05 – 0.06	−0.03	−0.09 – 0.02	0.01	−0.06 – 0.07	−0.20	−0.27 – −0.14
Neuroticism	−0.10	−0.24 – 0.03	−0.10	−0.22 – 0.03	0.04	−0.09 – 0.17	0.08	−0.05 – 0.22	−0.11	−0.25 – 0.02	−0.03	−0.13 – 0.07	−0.13*	−0.23 – −0.03
Conscientiousness	−0.28***	−0.42 – −0.15	−0.14*	−0.27 – −0.02	0.16**	0.04–0.29	0.08	−0.05 – 0.21	0.05	−0.09 – 0.18	0.04	−0.06 – 0.14	0.06	−0.04 – 0.16
Intra‐stress neuroticism*	−0.02	−0.06 – 0.03	0.00	−0.00 – 0.01	0.02	−0.03 – 0.08	−0.01	−0.07 – 0.04	0.01	−0.04 – 0.06	0.04	−0.02 – 0.10	−0.02	−0.08 – 0.03
Intra‐stress conscientiousness*	−0.04	−0.09 – 0.01	−0.08**	−0.14 – −0.02	−0.03	−0.09 – 0.03	0.01	−0.05 – 0.07	−0.06*	−0.12 – −0.00	0.00	−0.06 – 0.07	−0.03	−0.09 – 0.04
Alcohol (yes)	0.57***	0.41–0.73	0.22*	0.05 – 0.40	−0.37***	−0.55 – −0.19	−0.03	−0.21 – 0.15	−0.24**	−0.42 – −0.07	−0.18	−0.38– 0.02	−0.16	−0.35 – −0.03
Caffeine (yes)	0.11	−0.04 – 0.25	−0.13	−0.29 – 0.02	−0.22**	−0.38 – −0.06	−0.03	−0.19 – 0.13	−0.12	−0.28 – 0.03	−0.00	−0.16 – 0.16	−0.20*	−0.36 – −0.04

*Note*: 840 daily observations were clustered in 92 participants. *R* ^2^ conditional is effect size of the model including both fixed and random effects.

Abbreviations: CI (95%), 95% confidence interval; SE, sleep efficiency; SOL, sleep‐onset latency; TST, total sleep time; WASO, wake after sleep onset.

* *p* < 0.05;

** *p* < 0.01;

*** *p* < 0.001.

Neuroticism was significantly negatively (*β* = −0.13, *p* = 0.012) associated with subjective sleep quality, indicating that participants who were more neurotic reported worse sleep quality over the whole period (i.e. baseline and exam; Figure S2). Intra‐individual variation in stress levels, neuroticism and their interaction were not significantly associated with any of the actigraphic sleep variables (all *p* > 0.10).

**FIGURE 1:**
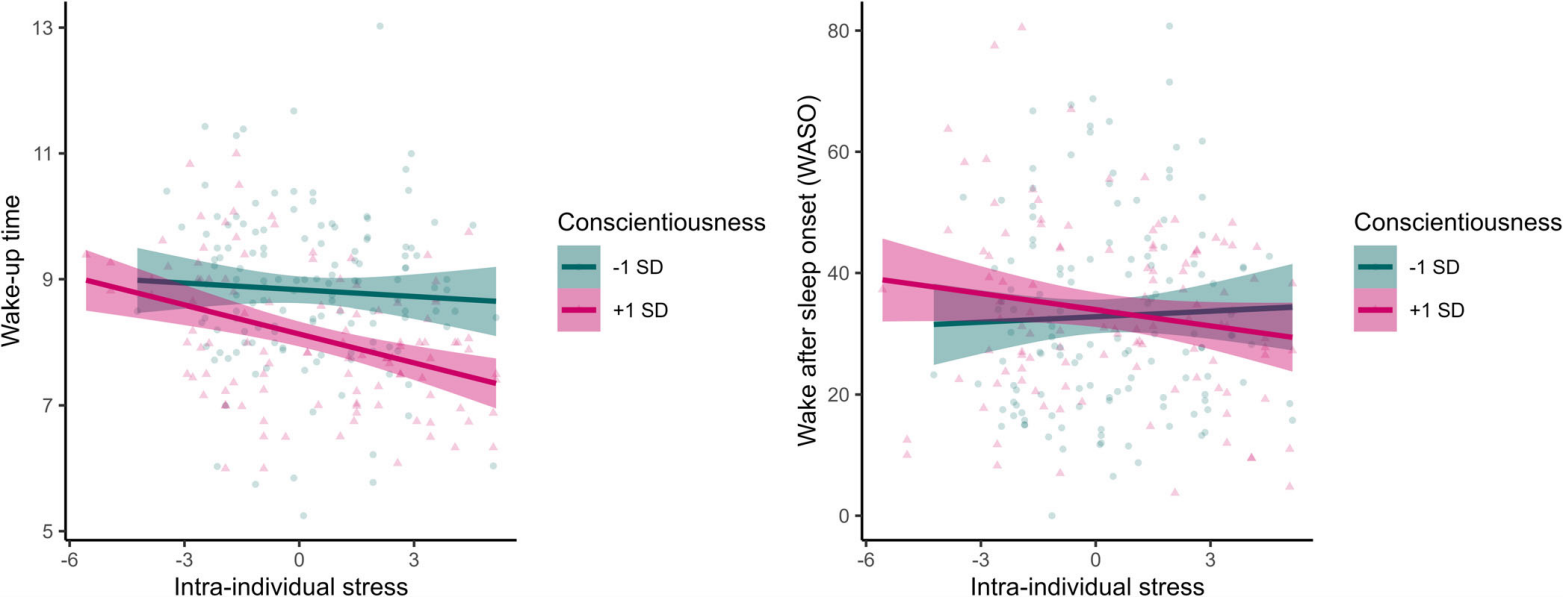
The effect of intra‐individual stress on timing (wake‐up time, left) and fragmentation (wake after sleep onset [WASO], right) of sleep differed between high and low conscientious participants.

Conscientiousness was significantly associated with the timing and duration of sleep, indicating that participants who were more conscientious went to bed earlier (*β* = −0.28, *p* < 0.001), woke up earlier (*β* = −0.14, *p* = 0.04) and had longer TST (*β* = 0.16, *p* = 0.01) over the whole period (Figure S2). In addition, conscientiousness significantly moderated the association between intra‐individual stress and WASO (*β* = −0.06, *p* = 0.04), and between intra‐individual stress and wake‐up time (*β* = −0.08, *p* = 0.008; Figure 1). For participants with high levels of conscientiousness (+ 1SD from the mean), the association between stress and WASO was negative, indicating that they had less fragmented sleep (WASO) in the nights following days when they experienced more stress than usual, while for participants with low levels of conscientiousness (−1SD from the mean), the association between stress and WASO was positive, indicating they had more fragmented sleep (WASO) in the night following a day with more stress than usual. Regarding wake‐up time, participants with high levels of conscientiousness (+ 1SD from the mean) had a stronger negative association between stress and wake‐up time than in people with low conscientiousness (−1SD from mean), indicating that participants with high conscientiousness wake‐up earlier when they experienced more stress than usual the day before (Figure 1). Conscientiousness was not significantly associated with the other sleep variables, including SE, SOL and subjective sleep quality (all *p* > 0.09).

### 3.4 Exploratory: Intra‐individual associations of sleep on stress the next day and the moderation by personality traits

The results of the exploratory multilevel models examining the temporal relationship of intra‐individual sleep on stress the next day and the moderation by personality are presented in the supplementary materials (Table S4). Participants experienced more stress the next day on days they went to bed (*β* = −0.11, *p* = 0.02) and woke up (*β* = −0.22, *p* = 0.001) earlier than usual. Neuroticism significantly moderated the association of intra‐individual variations in wake times on next‐day stress (*β* = −0.08, *p* = 0.02). Participants with high levels of neuroticism (+ 1SD from the mean) had a stronger negative association between wake‐up times and stress than in people with low neuroticism, indicating that participants with high neuroticism experience more stress the next day when they woke up earlier than usual. Conscientiousness significantly moderated the association of both intra‐individual variations in wake times (*β* = −0.14, *p* < 0.001) and bedtimes (*β* = −0.08, *p* = 0.03) on next‐day stress. Participants with high levels of conscientiousness (+ 1SD from the mean) had a stronger negative association between wake‐up times, bedtimes and stress than in people with low conscientiousness, indicating that participants with high conscientiousness experience more stress the next day when they woke up earlier and went to bed earlier than usual. Intra‐individual variation in the other sleep variables (TST, SE, WASO, SOL and sleep quality) was not associated with next‐day stress levels.

## 4 DISCUSSION

This study examined the temporal association of stress on sleep, and whether this relationship is moderated by the personality traits neuroticism and conscientiousness. Intra‐individual daily variations in stress were not associated with the following night's sleep timing, duration or fragmentation, implying that more stress during the day did not affect sleep the following night. Higher levels of conscientiousness were associated with earlier bed and wake times and a longer sleep duration. Participants with high levels of conscientiousness had less fragmented sleep and woke‐up earlier when they experienced more stress than usual. Neuroticism was only associated with daily subjective sleep quality, indicating that more neurotic participants reported worse sleep quality.

Averaged results over the exam and baseline period showed that reported daily stress was higher in the exam period compared with baseline, suggesting that our natural manipulation did indeed result in variation in stress levels. Subjective sleep quality was also lower during the exam period compared with baseline. Nevertheless, there was no difference in objective actigraphy measures of sleep between these periods. This is in line with a study in adolescents that also did not find differences between exam period and baseline in objective sleep (TST, SE or WASO; Dewald et al., 2014). Subjective and not objective sleep quality might therefore be mostly impacted during exam stress. Furthermore, when stress and sleep data were averaged over the whole period, there was no correlation between stress and the timing of sleep, duration or fragmentation. Participants who experienced over the whole period more stress, did report worse sleep quality, were more anxious and were more neurotic. These findings suggest that students who are more neurotic/anxious also report experiencing more stress and having worse sleep.

Our results did not indicate that more neurotic individuals reported worse sleep when stressed. Previous research findings are mixed. Cross‐sectional studies suggested that neuroticism would intensify the link between stress and sleep (Nédélec et al., 2021; Williams & Moroz, 2009), while others did not find a moderating effect of neuroticism on the daily association between rumination, negative affect and subjective sleep quality (Slavish et al., 2018). In our study, participants with higher levels of neuroticism did report worse daily subjective sleep quality and higher stress levels over the complete period, suggesting that neuroticism is primarily associated with subjective experiences (Zamani et al., 2022). Therefore, more neurotic students might benefit the most from sleep and coping with stress interventions, especially considering the association between neuroticism and worrying, anxiety and mental health (Muris et al., 2005), and the impact of poor sleep and stress on psychological well‐being (Kalmbach et al., 2018).

Conscientiousness moderated the association between intra‐individual stress and sleep fragmentation and wake‐up time, indicating that participants who were more conscientious had less fragmented sleep the night following a more stressful day. The association between earlier bed and wake‐time and higher conscientiousness is consistent with prior research indicating that conscientious individuals are more often morning types (Duggan et al., 2014). Conscientiousness is seen as a stress‐reducing trait characterized by self‐discipline and goal‐oriented behaviour (Leger et al., 2016). Moreover, individuals who are more conscientious have been found to utilize more effective coping strategies to deal with stress, this might be why their sleep was less fragmented after a stressful day (Penley & Tomaka, 2002). A speculative explanation, for our findings, might be that participants who are more conscientious got up earlier when experiencing more stress (e.g. due to exams) to have more time to prepare for their exams.

There was no bidirectional temporal association between stress and sleep in our study. A similar study in students also did not find an effect of stress on SE, WASO or SOL (Yap et al., 2020). Studies that did find an effect were often in working people (Buxton et al., 2016; Lee et al., 2017) or adolescents (Doane & Thurston, 2014). These groups might experience different types and intensities of stress. In addition, students might work extra hard around exams increasing their sleep drive and making it easier for them to fall asleep. Furthermore, previous research shows that students have good sleep hygiene knowledge, which they might apply during exam periods knowing the importance of sleep for memory and cognition (Felix et al., 2017). Another possible explanation why we did not find a temporal association between stress and sleep could be that we asked about previous day stress the following morning. Therefore, this measure of stress might have been less reliable as participants might have over‐reported their stress as we often remember the peak moments; or they might have under‐reported their stress as sleep aids overnight downregulation of stress so participants might have felt less stressed the following morning (Talamini et al., 2013). Moreover, previous research in university students showed that a shorter sleep duration due to stress was associated with specifically evening stress (Yap et al., 2020). Finally, stress has been associated with irregularity of sleep timing and duration in students (Kwon et al., 2022; Veeramachaneni et al., 2019). Therefore, timing and duration of sleep within an individual might vary a lot from night to night under stress, and could explain why we did not find a consistent effect of stress on the following night's sleep. However, our results did not show more variability in the sleep variables during the exam period. Future studies could focus on irregularities in sleep and use experience sampling method to capture stress levels at multiple moments during the day, including right before bedtime, and in addition assess bedtime rumination that might increase SOL.

This study has strong ecological validity, as participants were examined in their own environment using natural stressors (e.g. exam week). In addition, multiple components of sleep including objective and subjective measures were assessed, and compliance was high as almost all sleep diaries were filled in. Nonetheless, there were some limitations that are worth mentioning. First, a general limitation of actigraphy is the possible overestimation of sleep, specifically SOL and sleep duration, as sleep is inferred from a lack of motion during the night (i.e. lying still might incorrectly be classified as asleep). In addition, it lacks the ability to reveal the influence of stress on sleep architecture (Martin & Hakim, 2011). Second, our sample consisted of young healthy university students, all prone to the same type of academic stress. Thus, the generalizability of our findings may be restricted and not generalize to individuals experiencing other high stress situations for longer and steadier periods (e.g. patients with cancer or working adults with stressful jobs). Third, several exam periods were around daylight saving times (twice change to wintertime and once change to summertime), as we were dependent on the exam periods we could not change the time of our measurements. Nevertheless, changes in time due to daylight saving time might have impacted our sleep measures.

## 5 CONCLUSION

The utilization of objective assessments in examining the role of personality in the interplay between stress and sleep has the potential to yield novel insights. Students that were more conscientious had less fragmented sleep and earlier wake‐up times, following days when they experienced more stress than usual, while more neurotic students reported worse daily sleep quality. This study did not find a bidirectional temporal association between stress and sleep or a moderating role of neuroticism. For future studies, it would be interesting to include polysomnography to examine changes in the sleep architecture following more stressful days. For instance, studies have demonstrated alterations in sleep architecture, such as sleep spindles and slow‐wave sleep, following induced stress during a nap (Beck et al., 2022). To further disentangle the interplay between stress and sleep, objective markers of stress such as cortisol, heart rate variability or blood pressure could be assessed. The current findings suggest that the relationship between stress, sleep and personality is intertwined. Addressing one aspect of this relationship, such as managing stress or improving sleep quality, can have positive effects on the others.

## AUTHOR CONTRIBUTIONS

**Conny W. E. M. Quaedflieg:** Conceptualization; writing – review and editing. **Camilla Bossi:** Investigation; writing – review and editing; formal analysis. **Jessica Bruijel:** Conceptualization; investigation; writing – original draft; writing – review and editing; project administration; supervision; formal analysis.

## FUNDING INFORMATION

The authors have no financial involvement to disclose.

## CONFLICT OF INTEREST STATEMENT

The authors have no conflict of interest.

## Supporting information


**DATA S1** Supporting information.

## Data Availability

Data can be obtained via the Dutch Dataverse Network upon request: https://doi.org/10.34894/PV5QB7.
