# Supplementary material for: The moderating role of conscientiousness in the temporal association of stress on sleep
Source: J Sleep Res. 2024 Apr 29;33(6):e14224. doi: 10.1111/jsr.14224 (PMC11596995; doi:10.1111/jsr.14224)
Supplement: Supplementary file 1 — DATA S1 Supporting information. [file JSR-33-e14224-s001.docx]

# **Supplementary materials**

| **Table S1.** Demographic and questionnaire results for sleep quality, anxiety, personality and perceived stress (N=92) | | |
| --- | --- | --- |
| **Variable** | **Mean ± SD or N (%)** | **Range** |
| Current employment (yes) | 28 (30.4%) |  |
| Living situation: |  |  |
| Living alone | 22 (23.9%) |  |
| Living with partner | 11 (12.0%) |  |
| Living with roommates | 48 (52.2%) |  |
| Living with family | 11 (12.0%) |  |
| Sleep quality (PSQI) | 5.8 ± 2.5 | 2-15 |
| *Clinically significant (>5)* | 42 (45.7%) |  |
| Trait anxiety (STAI) | 45.4 ± 10.0 | 24-71 |
| *Clinically significant (>45)* | 44 (47.8%) |  |
| Personality (BFI) |  |  |
| Extraversion | 27.6 ± 6.4 | 11-40 |
| Agreeableness | 35.0 ± 4.9 | 23-45 |
| Neuroticism | 25.8 ± 5.9 | 10-38 |
| Openness | 37.1 ± 5.2 | 22-47 |
| Conscientiousness | 31.3 ± 5.7 | 16-45 |
| Perceived stress (PSS) | 19.1 ± 5.1 | 8-34 |
| *Note*: PSS (N=70). SD, standard deviation; PSQI, Pittsburgh Sleep Quality Index; STAI, State-Trait Anxiety Inventory; BFI, Big Five Inventory; PSS, Perceived Stress Scale. | | |

*Average sleep data*

Over the whole measurement period participants slept on average 7.0 hours per night (SD, 0.8), with a bedtime ranging from 22:00 to 02:54, and a wake time ranging from 05:13 to 10:21. Moreover, participants had an average SOL of 15 minutes, SE of 88 % and WASO of 33 minutes. For young adults sleep duration between 7 and 9 hours is recommend and a sleep efficiency higher the 85% is considered good. This indicates that overall participants in our study did not have poor sleep. However, some participants were below these recommendations, thus not getting the recommended hours of sleep.

*Sleep in correlation with stress, neuroticism, conscientiousness and anxiety*

To compare the current results with previous studies, we calculated correlations between the averaged sleep variables over the complete period and stressed averaged over the whole period, neuroticism, conscientiousness, anxiety and perceived stress (see table S2). After correction for multiple comparisons only higher levels of daily stress and anxiety were correlated with worse daily subjective sleep quality (all *p*’s <.001). Higher levels of neuroticism, and anxiety and worse subjective sleep quality and more perceived stress were all correlated with higher levels of reported stress (all *p*’s <.001). Higher levels of conscientiousness were associated with earlier bedtimes. Stress, neuroticism, anxiety and daily subjective sleep quality (diary) were not significantly correlated with any of the other sleep variables such as bedtimes, SOL, SE, WASO, or TST.

| **Table S2**. Correlations between actigraphic sleep variables, diary stress and sleep quality variables averaged over the measurement period and anxiety, personality, and perceived stress (N=92). | | | | | | | | | | | | | | | | |
| --- | --- | --- | --- | --- | --- | --- | --- | --- | --- | --- | --- | --- | --- | --- | --- | --- |
|  | **Bedtime** | | **Wake time** | | **Sleep onset latency** | | **Sleep efficiency** | | **Wake after sleep onset** | | **Total sleep time** | | **Sleep Quality (diary)** | | **Total stress** | |
|  | *r* | *p* | *r* | *p* | *r* | *p* | *r* | *p* | *r* | *p* | *r* | *p* | *r* | *p* | *r* | *p* |
| **Sleep Quality (diary)** | -0.02 | .85 | 0.09 | .39 | -0.06 | .60 | 0.14 | .19 | -0.02 | .83 | 0.11 | .27 |  |  | -0.36 | **<.001** |
| **Total Stress** | -0.08 | .47 | -0.22 | *.04* | 0.15 | .15 | -0.12 | .27 | -0.05 | .65 | -0.12 | .26 | -0.36 | **<.001** |  |  |
| **Sleep quality (PSQI)** | 0.14 | .20 | -0.21 | *.04* | 0.11 | .30 | -0.12 | .26 | -0.21 | *.04* | -0.35 | **<.001** | -0.53 | **<.001** | 0.33 | **<.001** |
| **Anxiety (STAI)** | -0.11 | .30 | -0.21 | *.04* | -0.09 | .38 | 0.04 | .72 | -0.11 | .28 | -0.06 | .57 | -0.35 | **<.001** | 0.55 | **<.001** |
| **Neuroticism (BFI)** | -0.10 | .32 | -0.15 | .16 | -0.05 | .62 | 0.10 | .35 | -0.18 | .09 | 0.02 | .88 | -0.29 | *<.01* | 0.41 | **<.001** |
| **Conscientiousness (BFI)** | -0.37 | **<.001** | -0.21 | *.04* | 0.09 | .40 | 0.12 | .25 | 0.07 | .51 | 0.23 | *.03* | 0.13 | .22 | -0.23 | *.03* |
| **Perceived stress (PSS)** | 0.23 | .06 | 0.06 | .64 | -0.11 | .38 | 0.05 | .68 | -0.23 | .05 | -0.16 | .18 | -0.31 | *<.01* | 0.51 | **<.001** |
| *Note:* Only the bold *p*-values were significant after Bonferroni correction for multiple comparison (*p*< .006). PSS (N=70). PSQI, Pittsburgh Sleep Quality Index; STAI, State-Trait Anxiety Inventory; BFI, Big Five Inventory; PSS, Perceived Stress Scale. | | | | | | | | | | | | | | | | |


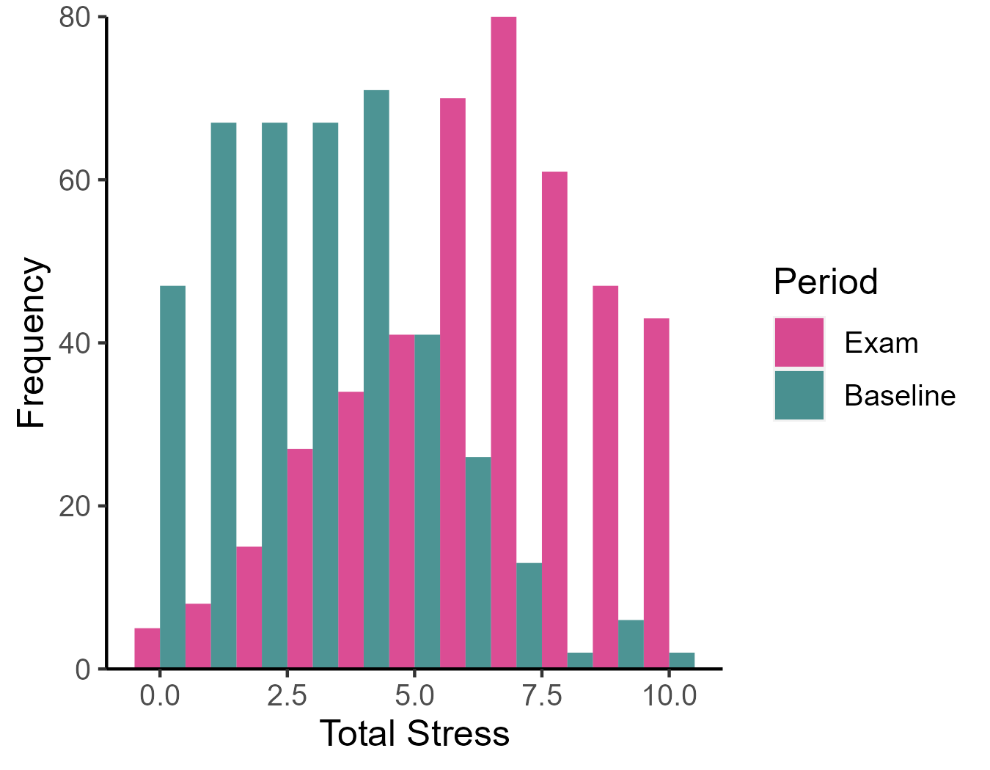


**Figure S1.** The variability of total stress levels in the baseline (green) and exam period (pink).

*Variability in sleep*

There was more variability during the baseline period in bedtimes and wake-up times compared to the exam period (Table S3). Nonetheless, there was more variability in subjective sleep quality during the exam period compared to baseline.

Only variability in SE was correlated with average stress levels, however, this was not significant after Bonferroni correction (*r*=0.22, *p*=.04). There was no significant correlation between variability in the other sleep variables and average stress over the whole period (all *p*’s >.10).

| **Table S3.** Variability in the sleep variables between the baseline and exam period (N=87*). | | | | | | |
| --- | --- | --- | --- | --- | --- | --- |
|  | **Baseline** | | **Exam period** | | ***Statistics*** |  |
| **Variables** | Mean ± SD | Range | Mean ± SD | Range |  |  |
| Bed time | 1.0 ± 0.6 | 0.0-2.9 | 0.8 ± 0.5 | 0.2-2.1 | **t(1, 86)= -2.07, *p* = .04** |  |
| Wake-up time | 1.0 ± 0.4 | 0.1-2.0 | 0.9 ± 0.5 | 0.2-4.1 | **t(1, 86)= -2.74, *p* =.007** |  |
| Time in bed | 73.1 ± 40.1 | 10.0-207.5 | 72.1 ± 58.9 | 17.3-367.8 | t(1, 86)= -0.51, *p* = .61 |  |
| Total sleep time (TST) | 10.7 ± 6.1 | 1.0-29.1 | 9.8 ± 5.9 | 1.1-41.6 | t(1, 86)= -1.87, *p* = .06 |  |
| Sleep onset latency (SOL) | 12.8 ± 9.1 | 0.0-43.7 | 12.4 ± 7.9 | 0.0-32.4 | t(1, 86)= -0.34, *p* = .73 |  |
| Sleep efficiency (SE) | 3.9 ± 2.2 | 0.7-10.3 | 3.7 ± 1.9 | 0.5-8.5 | t(1, 86)= -0.67, *p* = .50 |  |
| Wake after sleep onset | 59.9 ± 30.6 | 8.9-155.0 | 53.4 ± 33.9 | 5.3–204.1 | t(1, 86)= -4.32, *p* = .19 |  |
| Subjective sleep quality | 0.6 ± 0.3 | 0.0-1.4 | 0.7 ± 0.3 | 0.0-1.8 | **t(1, 86)= 2.19, *p* = .03** |  |
| * Five participants did not have enough datapoints for the exam or baseline period to calculate the variability and were thus not included in the analyses. | | | | | |  |


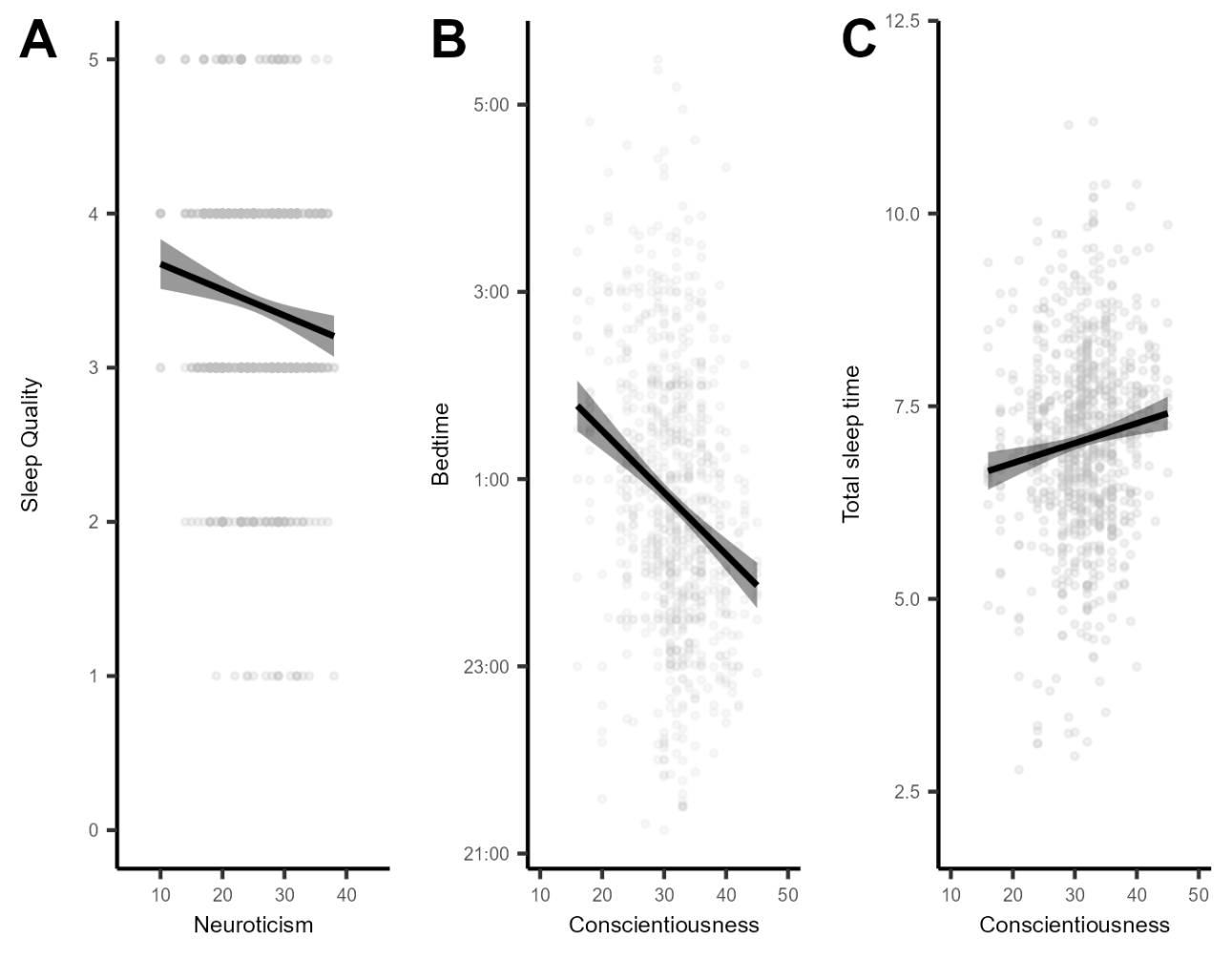


**Figure S2.** Association between neuroticism and sleep quality (A), conscientiousness and bedtime (B), and conscientiousness and total sleep time (C).

| **Table S4.** Results from multilevel models of intra-individual association of sleep on stress including the moderation by personality (N=92). | | | | | |
| --- | --- | --- | --- | --- | --- |
|  | | **Full model** | | **Predictor** | |
|  | | *R^2 Conditional^* |  | *Beta* | *CI (95%)* |
| **Model Bed time** | | 0.21 | T=3.35*, p=.*001 |  |  |
|  | Intra-bedtime |  |  | **-0.11*** | -0.18 – -0.05 |
|  | Neuroticism |  |  | **0.22***** | 0.12 – 0.32 |
|  | Conscientiousness |  |  | **-0.10*** | -0.20 – 0.00 |
|  | Intra-bed time*Neuroticism |  |  | -0.06 | -0.12 – 0.01 |
|  | Intra-bed time* Conscientiousness |  |  | **-0.08*** | -0.15 – -0.01 |
| **Model Wake time** | | 0.25 | T=3.69, *p*<.001 |  |  |
|  | Intra-wake time |  |  | **-0.22**** | -0.28 – -0.15 |
|  | Neuroticism |  |  | **0.20***** | 0.11 –  0.30 |
|  | Conscientiousness |  |  | **-0.11*** | -0.21 – -0.01 |
|  | Intra-wake time*Neuroticism |  |  | **-0.08*** | -0.14 – -0.01 |
|  | Intra-wake time* Conscientiousness |  |  | **-0.14***** | -0.20 – -0.07 |
| **Total sleep time** | | 0.20 | T=3.35, *p*=.001 |  |  |
|  | Intra-TST |  |  | -0.11 | -0.18 – -0.05 |
|  | Neuroticism |  |  | **0.21***** | 0.11 –  0.31 |
|  | Conscientiousness |  |  | **-0.10*** | -0.20 – -0.00 |
|  | Intra-TST*Neuroticism |  |  | -0.00 | -0.07 –  0.07 |
|  | Intra-TST* Conscientiousness |  |  | -0.05 | -0.11 –  0.02 |
| **Sleep efficiency** | | 0.19 | T=3.25, *p*=.001 |  |  |
|  | Intra-SE |  |  | 0.01 | -0.06 – 0.08 |
|  | Neuroticism |  |  | **0.22***** | 0.12 – 0.31 |
|  | Conscientiousness |  |  | -0.10 | -0.20 – 0.00 |
|  | Intra-SE*Neuroticism |  |  | 0.01 | -0.06 – 0.08 |
|  | Intra-SE* Conscientiousness |  |  | 0.01 | -0.05 – 0.07 |
| **Wake after sleep onset** | | 0.20 | T=3.29, *p*=.001 |  |  |
|  | Intra-WASO |  |  | -0.08 | -0.15 – -0.01 |
|  | Neuroticism |  |  | **0.21***** | 0.11 –  0.31 |
|  | Conscientiousness |  |  | **-0.10*** | -0.20 –  0.00 |
|  | Intra-WASO*Neuroticism |  |  | 0.02 | -0.05 –  0.09 |
|  | Intra-WASO* Conscientiousness |  |  | -0.03 | -0.10 –  0.03 |
| **Sleep onset latency** | | 0.19 | T=3.25, *p*=.001 |  |  |
|  | Intra-SOL |  |  | 0.03 | -0.04 – 0.10 |
|  | Neuroticism |  |  | **0.22***** | 0.12 – 0.32 |
|  | Conscientiousness |  |  | -0.10 | -0.20 – 0.00 |
|  | Intra-SOL*Neuroticism |  |  | 0.00 | -0.07 – 0.07 |
|  | Intra-SOL* Conscientiousness |  |  | -0.01 | -0.07 – 0.05 |
| **Sleep quality** | | 0.24 | T=0.95, *p*=.35 |  |  |
|  | Sleep quality |  |  | -0.24 | -0.31 – -0.17 |
|  | Neuroticism |  |  | **0.20**** | 0.10 – 0.30 |
|  | Conscientiousness |  |  | -0.09 | -0.19 – 0.00 |
|  | Sleep quality*Neuroticism |  |  | -0.07 | -0.14 – 0.00 |
|  | Sleep quality* Conscientiousness |  |  | -0.04 | -0.10 – 0.03 |
| TST, Total sleep time; SE, Sleep efficiency; WASO, Wake after sleep onset; SOL, Sleep onset latency; CI (95%), 95% Confidence Interval. R^2 conditional^ is effect size of the model including both fixed and random effects. **p*<.05 ***p*<.01 ****p*<.001 | | | | | |
